# Mutational analysis of the tyrosine kinome in serous and clear cell endometrial cancer uncovers rare somatic mutations in *TNK2* and *DDR1*

**DOI:** 10.1186/1471-2407-14-884

**Published:** 2014-11-26

**Authors:** Meghan L Rudd, Hassan Mohamed, Jessica C Price, Andrea J O’Hara, Matthieu Le Gallo, Mary Ellen Urick, NISC Comparative Sequencing Program, Pedro Cruz, Suiyuan Zhang, Nancy F Hansen, Andrew K Godwin, Dennis C Sgroi, Tyra G Wolfsberg, James C Mullikin, Maria J Merino, Daphne W Bell

**Affiliations:** Cancer Genetics Branch, National Human Genome Research Institute, National Institutes of Health, Bethesda, MD 20892 USA; NIH Intramural Sequencing Center, National Institutes of Health, Bethesda, MD 20892 USA; Genome Technology Branch, National Human Genome Research Institute, National Institutes of Health, Bethesda, MD 20892 USA; Department of Pathology and Laboratory Medicine, University of Kansas Medical Center, Kansas, KS 66160 USA; Molecular Pathology Unit and Center for Cancer Research, Massachusetts General Hospital, 149 13th Street, Charlestown, MA 02129 USA; Center for Cancer Research, National Cancer Institute, National Institutes of Health, Bethesda, MD 20892 USA

**Keywords:** Endometrial, Cancer, Mutation, TNK2, ACK1, DDR1, Copy number, Tyrosine kinase, Tyrosine kinome

## Abstract

**Background:**

Endometrial cancer (EC) is the 8^th^ leading cause of cancer death amongst American women. Most ECs are endometrioid, serous, or clear cell carcinomas, or an admixture of histologies. Serous and clear ECs are clinically aggressive tumors for which alternative therapeutic approaches are needed. The purpose of this study was to search for somatic mutations in the tyrosine kinome of serous and clear cell ECs, because mutated kinases can point to potential therapeutic targets.

**Methods:**

In a mutation discovery screen, we PCR amplified and Sanger sequenced the exons encoding the catalytic domains of 86 tyrosine kinases from 24 serous, 11 clear cell, and 5 mixed histology ECs. For somatically mutated genes, we next sequenced the remaining coding exons from the 40 discovery screen tumors and sequenced all coding exons from another 72 ECs (10 clear cell, 21 serous, 41 endometrioid). We assessed the copy number of mutated kinases in this cohort of 112 tumors using quantitative real time PCR, and we used immunoblotting to measure expression of these kinases in endometrial cancer cell lines.

**Results:**

Overall, we identified somatic mutations in *TNK2* (tyrosine kinase non-receptor, 2) and *DDR1* (discoidin domain receptor tyrosine kinase 1) in 5.3% (6 of 112) and 2.7% (3 of 112) of ECs. Copy number gains of *TNK2* and *DDR1* were identified in another 4.5% and 0.9% of 112 cases respectively. Immunoblotting confirmed TNK2 and DDR1 expression in endometrial cancer cell lines. Three of five missense mutations in *TNK2* and one of two missense mutations in *DDR1* are predicted to impact protein function by two or more *in silico* algorithms. The TNK2^P761Rfs*72^ frameshift mutation was recurrent in EC, and the DDR1^R570Q^ missense mutation was recurrent across tumor types.

**Conclusions:**

This is the first study to systematically search for mutations in the tyrosine kinome in clear cell endometrial tumors. Our findings indicate that high-frequency somatic mutations in the catalytic domains of the tyrosine kinome are rare in clear cell ECs. We uncovered ten new mutations in *TNK2* and *DDR1* within serous and endometrioid ECs, thus providing novel insights into the mutation spectrum of each gene in EC.

**Electronic supplementary material:**

The online version of this article (doi:10.1186/1471-2407-14-884) contains supplementary material, which is available to authorized users.

## Background

Endometrial carcinomas (ECs) arise from the inner epithelial lining of the uterus and can be classified into a number of discrete histological subtypes (reviewed in [1]). Endometrioid endometrial carcinomas (EECs) represent the vast majority of diagnosed cases [1]. They are generally estrogen-dependent tumors that are associated with a number of well-established epidemiological risk factors that lead to unopposed estrogen exposure including obesity, nulliparity, early age at menarche, and late age at menopause [2]. Most EECs are detected at an early clinical stage when surgery or surgery with adjuvant radiotherapy can often be curative [3, 4].

Serous and clear cell ECs are high-grade tumors that are rare at diagnosis but are clinically aggressive and contribute substantially to mortality from endometrial cancer (reviewed in [5]). For example, in a large retrospective study of 5,694 cases of endometrial cancer in the US, serous and clear cell tumors together represented 13% of diagnoses but accounted for 47% of deaths [6]. Historically, serous and clear cell ECs are considered to be estrogen-independent tumors with no well-established epidemiological risk factors other than increasing age [7, 8]. However, a recent large epidemiological study has suggested that increased body mass index may be a risk factor for serous endometrial carcinomas [9]. Current therapeutic approaches to treat patients with serous or clear cell ECs are variable but generally include surgery and adjuvant chemotherapy and/or radiotherapy [10, 11].

Alternative therapeutic options are being sought for patients with serous or clear cell EC and for patients with advanced-stage or recurrent endometrioid EC. Rationally-designed therapeutics targeting tyrosine kinases can be clinically efficacious against tumors that have somatically mutated, amplified, or rearranged the target kinase, and which are dependent on the aberrant kinase-mediated signaling for their survival [12–17]. Recently, the tyrosine kinase gene family has been sequenced in 133 serous ECs, 329 endometrioid ECs, 53 ECs of unspecified histology, and 13 mixed histology ECs either by targeted sequencing of the tyrosine kinome [18], or by comprehensive sequencing of all protein-encoding genes including the tyrosine kinome [19–24]. However, it has been estimated that at least 500 tumors of a given histology need to be sequenced to provide adequate statistical power to reliably detect mutations occurring at a frequency of at least 3% in a particular histotype [25]. Therefore, sequencing tyrosine kinase genes in additional serous ECs may shed further insights into the frequency and spectrum of mutations in potentially druggable targets in this clinically aggressive subtype. Moreover, the lack of a systematic search for mutations in the tyrosine kinome of clear cell ECs merits such an analysis for this histological subtype.

Here, we performed a mutation discovery screen to determine the incidence of somatic mutations in the catalytic domains of 86 tyrosine kinases in a series of 24 primary serous, 11 clear cell, and 5 mixed (serous-endometrioid) histology ECs. Somatically mutated genes were then resequenced from another 72 ECs, and evaluated for copy number alterations in all 112 tumors. We report low-frequency somatic mutations and copy number gains of the *TNK2* (tyrosine kinase non-receptor, 2) and *DDR1* (discoidin domain receptor tyrosine kinase 1) kinases among the three major histological subtypes of EC.

## Methods

### Ethics statement

The NIH Office of Human Subjects Research determined that this research activity was exempt from Institutional Review Board review.

### Clinical specimens

Anonymized, fresh-frozen, primary tumor tissues and matched histologically normal tissues were obtained from the Cooperative Human Tissue Network (100 cases), which is funded by the National Cancer Institute, or from the Biosample Repository at Fox Chase Cancer Center, Philadelphia PA (1 case). DNAs from another 11 cases of fresh-frozen tissue, including all five mixed histology (endometrioid-serous) cases (Additional file 1), were purchased from Oncomatrix. To the best of our knowledge, the mixed-histology tumor tissues were not macrodissected to separate individual histological components prior to DNA extraction by Oncomatrix. The entire cohort of 112 cases consisted of 45 serous, 21 clear cell, 41 endometrioid, and 5 mixed histology ECs. The endometrioid cases consisted of grade 1 (n = 26), grade 2 (n = 12), grade 2/3 (n = 1), and grade 3 (n = 2) tumors (Additional file 1). All primary tumor tissues were collected prior to treatment. For tumor tissues (n = 100) procured from CHTN, a hematoxylin and eosin (H&E) stained section was cut from each tumor specimen and reviewed by a pathologist to verify histology and to delineate regions of tissue with a tumor cell content of ≥70%.

### Nucleic acid isolation

Genomic DNA was isolated from macrodissected tissue with greater than 70% tumor cellularity using the Puregene kit (Qiagen).

### Identity testing

Paired tumor-normal DNA samples were genotyped using the Coriell Identity Mapping kit (Coriell). Genotyping fragments were size separated on an ABI-3730*xl* DNA analyzer (Applied Biosystems). Alleles were scored using GeneMapper software.

### Primer design, PCR amplification, nucleotide sequencing and variant calling

M13-tailed primer pairs (Additional file 2) were designed to target 577 of 591 exons that encode the catalytic domains of the 86 protein tyrosine kinases (Additional file 3), using previously published methods [26]. Sequence constraints precluded the design of primers for 14 of 591 exons. Primers were also designed to target the exons that encode the exonuclease domain (exons 3 to 13) of *POLE* (polymerase (DNA directed), epsilon, catalytic subunit) and are available on request. PCR amplification conditions are available upon request. Bidirectional Sanger sequencing of PCR products and subsequent nucleotide variant calling were performed as previously described [27]. Variant positions were cross-referenced to the dbSNP (Build 129) database to annotate and exclude known germline variants. To determine whether novel variants were somatic mutations or germline variants, the appropriate tumor DNA and matched normal DNA were re-amplified in an independent PCR followed by sequence analysis of the variant position. Primers used in the secondary screen of *TNK2* and *DDR1* are provided in Additional file 4.

### Quantitative real-time PCR

Predesigned primers targeting *TNK2* (VPH103-1002824A), *DDR1* (VPH106-0859748A) and *B2M* (beta-2-microglobulin) (VPH115-0515670A) were purchased from SABiosciences (Qiagen). Reactions were assembled to contain either Taqman control genomic DNA (Applied Biosystems) or 2 ng of tumor genomic DNA, 2 μl of primers (diluted 1:4), 3.5 μl SYBR Green Rox qPCR mastermix (Qiagen), to a final 10 μl reaction volume. qPCR was preformed on a ABI 7900 HT Fast Real-Time PCR System (Applied Biosystems) with the following cycle conditions: 50°C for 2 min, 95°C for 10 min, and 40 cycles of 95°C for 15 sec and 60°C for 1 min. A standard curve was generated with Taqman control genomic DNA, to permit a determination of the absolute quantitation using SDS 2.4 software (Applied Biosystems). For each experiment, tumor samples were assayed in triplicate for the target gene and control gene (*B2M*). For each sample, the mean quantity of each target gene was normalized to the mean quantity of *B2M*. For tumors displaying copy number gains (defined here as a ≥3-fold increase of the target gene compared to *B2M*), the matched normal DNAs were analyzed to confirm that the copy number gain was somatic. Three independent experiments were performed for each tumor and normal pair. The fold change in somatic copy number was determined by dividing the normalized mean quantity of the target gene in the tumor sample by the normalized mean quantity of the target gene in the matched normal sample. In addition, a 2-tailed Student *t*-test was used to calculate statistical significance.

### Estimation of statistical power of study design

The estimated power to detect one gene mutation in a set of 40 tumors was calculated as 1 - (1-X)^40, where X is the actual fraction of tumors with a mutation in that gene (Additional file 5).

### Cell lines and immunoblotting

Serous endometrial cancer cell lines (ARK1 and ARK2) were kindly provided by Dr. Alessandro Santin (Yale School of Medicine). RL-95-2, HEC1A, HEC1B, KLE were obtained from the American Type Culture Collection, or the National Cancer Institute’s Developmental Therapeutics Program. RL95-2 was established from a grade 2 moderately differentiated adenosquamous carcinoma of the endometrium [28], KLE was established from a poorly differentiated endometrial carcinoma [29], HEC1A was established from a human moderately differentiated endometrial adenocarcinoma [30, 31], and HEC1B is a sub-line of HEC1A [31, 32]. Cells were washed in phosphate-buffered saline then lysed with ice-cold RIPA buffer (Thermo Scientific) containing 1 mM Na-orthovanadate, 10 mM NaF, and 1X protease inhibitor cocktail (Roche). Lysates were centrifuged and proteins were quantitated with the Bio-Rad protein assay (Bio-Rad 500–0006). Equal amounts (μg) of the cleared lysate were denatured at 95°C in 2X SDS sample buffer (Sigma) prior to SDS-PAGE and transfer to PVDF membranes (Bio-Rad). Primary and HRP-conjugated secondary antibodies were: αDDR1 (Cell Signaling), αTNK2 (Upstate), αβ-Actin (Sigma), goat anti-mouse HRP (Cell Signaling), and goat anti-rabbit HRP (Cell Signaling). Immunoreactive proteins were visualized with enhanced chemiluminescence (Pierce).

## Results

### The *TNK2*and *DDR1*tyrosine kinases are somatically mutated in endometrial carcinomas

In a mutation discovery screen, we sequenced 577 exons that encode the catalytic domains of 86 tyrosine kinases (Additional file 3), from 24 serous, 11 clear cell, and 5 mixed (serous/endometrioid) histology endometrial carcinomas. We selectively sequenced the catalytic domain of each kinase because this domain can be preferentially mutated in other cancers [12, 15, 33]. For a gene that has kinase domain mutations at an actual frequency of 10%, we estimate that a discovery screen of 24 serous tumors has 92.0% statistical power to observe at least one mutation (Additional file 5). For a discovery screen of 11 clear cell tumors and 5 mixed histology tumors the corresponding statistical power is estimated to be 68.6% and 40.9% respectively (Additional file 5). Six serous tumors (T27, T33, T45, T56, T65, T75) in our discovery screen were previously subjected to whole exome sequencing [19].

**Figure 1:**
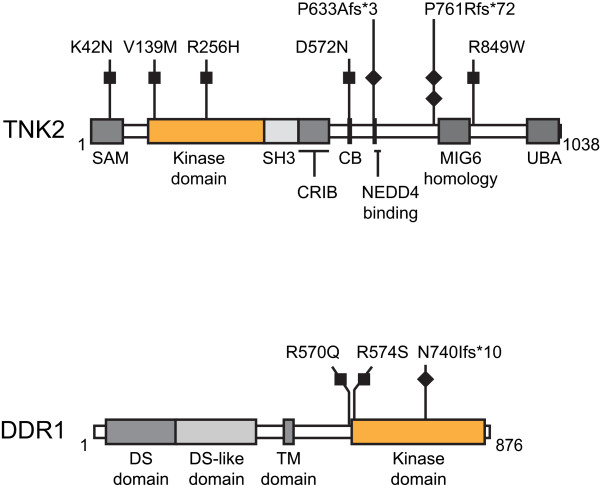
**Localization of nonsynonymous, somatic mutations in TNK2 and DDR1 relative to important functional domains of the proteins.** All the somatic mutations were uncovered in primary endometrial tumors. Individual missense mutations (black boxes) are distinguished from frameshift mutants (black diamonds). Abbreviations: CB, clathrin binding site; CRIB, Cdc42/Rac interactive binding; DS, discoidin; SAM, sterile alpha motif; SH3, Src Homology 3; TM, transmembrane; UBA, ubiquitin associated.

We obtained high quality sequence data for 84% (11.8 Mb) of targeted bases (14.1 Mb). After excluding known germline variants, there were 24 nucleotide variants that represented potential somatic mutations. Sequencing of the matched normal DNA revealed that two of the 24 variants were *bona fide* nonsynonymous somatic mutations. The somatic mutations occurred in *TNK2* (Tyrosine kinase non-receptor protein 2) and *DDR1* (Discoidin domain receptor tyrosine kinase 1). We therefore extended our analysis of *TNK2* and *DDR1* to sequence the remaining coding exons from the 40 tumors in the discovery screen and to sequence all coding exons of *TNK2* and *DDR1* from another 72 primary endometrial tumors consisting of 10 clear cell, 21 serous, and 41 endometrioid tumors*.* The secondary screen revealed nine additional nonsynonymous somatic mutations localizing to the catalytic and non-catalytic domains of the encoded proteins (Figure 1, Additional file 6, Additional file 7).

**Figure 2:**
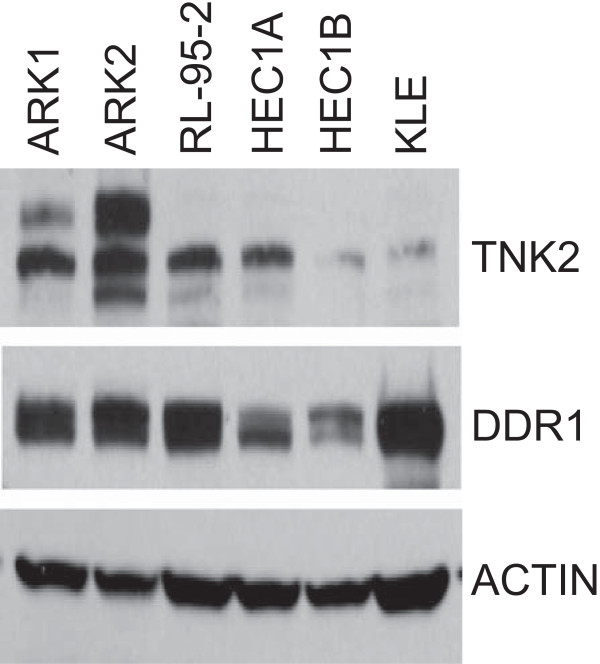
**TNK2 and DDR1 are expressed in endometrial cancer cell lines.** Immunoblots showing expression of the TNK2 and DDR1 proteins in a panel of endometrial cancer cell lines. Actin served as a loading control.

Overall, among the 112 tumors in this study, *TNK2* was somatically mutated in 2.2% (1 of 45) of serous, 4.8% (1 of 21) of clear cell, 7.3% (3 of 41) of endometrioid, and 20% (1 of 5) of mixed histology endometrial tumors. *DDR1* was somatically mutated in 4.4% (2 of 45) of serous tumors and in 2.4% (1 of 41) of endometrioid tumors (Table 1). Of the three endometrioid tumors that harbored somatic *DDR1* or *TNK2* mutations, two cases (T88 and T117) were grade 1 and one case (T131) was grade 3. Overall, there was no significant difference in the frequency of *TNK2/DDR1* mutations between low/intermediate-grade and high-grade endometrioid ECs; 2 of 38 (5.3%) low/intermediate-grade (grade 1 or grade 2) endometrioid ECs had a *TNK2* or *DDR1* mutation compared with 1 of 3 (33.3%) high-grade (grade 2/3 or 3) endometrioid ECs (*P* = 0.2086). The TNK2^D572N^, TNK2^R849W^, TNK2^R256H^, and DDR1^R570Q^ missense mutants are predicted, by at least two *in silico* algorithms, to impact the function of the encoded proteins (Table 1). Immunoblotting confirmed that TNK2 and DDR1 are endogenously expressed in endometrial cancer cells (Figure 2).

**Table 1:** **Somatic mutations of** ***TNK2*** **, and** ***DDR1*** **identified among 112 primary ECs**

Gene	Tumor ID	Histology and grade (G)	Nucleotide change	Amino acid change	***In silico***functional predictions		
					Mutation assessor	SIFT	Polyphen v2
*TNK2*	T3^a,b^	Serous	c.C1887_1888ins C	p.P633Afs*3	-	-	-
	T3^a,b^	Serous	c.G1714A	p.D572N	Low	Affects function	Probably damaging
	T15^a^	Mixed	c.G415A	p.V139M	Low	Affects function	Possibly damaging
	T77^c^	Clear cell	c.C2545T	p.R849W	Low	Affects function	Probably damaging
	T88^c^	Endometrioid (G1)	c.G767A	p.R256H	High	Affects function	Probably damaging
	T88^c^	Endometrioid (G1)	c.G126T	p.K42N	Medium	Tolerated	Probably damaging
	T117^c^	Endometrioid (G1)	c.2276 del C	p.P761Rfs*72	-	-	-
	T131	Endometrioid (G3)	c.2276 del C	p.P761Rfs*72	-	-	-
*DDR1*	T3^a,b^	Serous	c.C1720A	p.R574S	Low	Tolerated	Benign
	T79	Serous	c.G1709A	p.R570Q	Low	Affects function	Probably damaging
	T117^c^	Endometrioid (G1)	c.2216delA	p.N740Ifs*10	-	-	-

Transcript accession numbers: *TNK2* (Ensembl ID ENST00000392400), *DDR1* (Ensembl ID ENST00000454612). Protein accession numbers: TNK2 (CCDS33928), DDR1 (CCDS4690). G: Grade.

^a^Case no. T3 is also known as OM-1323, T15 is also known as OM-1529.

^b^ *POLE*-mutated.

^c^MSI-positive tumors, as reported previously [19].

*Denotes the position of a new stop codon introduced by the corresponding frameshift (fs) mutation.

### Increased copy number of *TNK2*and *DDR1*in endometrial carcinoma

We next used quantitative real-time PCR to determine whether *TNK2* or *DDR1* were affected by copy number alterations among the 112 endometrial tumors in this study. Somatic copy number increases of *TNK2* were detected in 8.9% (4 of 45) of serous tumors and in 2.4% (1 of 41) of endometrioid tumors, but in none of the clear cell tumors (Table 2). The single endometrioid tumor displaying a copy number gain of *TNK2* was a grade 2 tumor. Somatic copy number increases involving *DDR1* were detected in 2.2% (1 of 45) of serous tumors (Table 2). For each gene, tumors that displayed copy number alterations were distinct from tumors that had somatic mutations (Additional file 8). Considering mutations and copy number alterations together, *TNK2* was somatically altered in 11.1% (5 of 45) of serous, 4.8% (1 of 21) of clear cell, 9.8% (4 of 41) of endometrioid, and 20% (1 of 5) of mixed histology tumors and *DDR1* was somatically altered in 6.7% (3 of 45) of serous and 2.4% (1 of 41) of endometrioid tumors but not in clear cell or mixed histology tumors (Additional file 8).

**Table 2:** **Copy number gains of** ***TNK2*** **and** ***DDR1*** **among 112 primary ECs**

Gene	Tumor ID	Histology	Fold Increase in somatic copy number	p-value^§^
*TNK2*	T25	Serous	3.911	0.0000426
	T50	Serous	3.416	0.0018339
	T66	Serous	5.065	0.0185581
	T83	Serous	5.968	0.0171938
	T105	Endometrioid (G2)	3.374	0.0006604
*DDR1*	T23	Serous	3.019	0.0005727

^**§**^2-tailed Student *t*-test.

G: Grade.

Copy number gains of *TNK2* and *DDR1* could reflect either targeted gene amplification of these kinases or gain of a multigenic genomic region encompassing these genes. To discriminate between these two possibilities, we interrogated the copy number status of *TNK2, DDR1*, and their flanking genes within The Cancer Genome Atlas (TCGA) catalogue of somatic alterations in serous and endometrioid ECs [21], via the cBIO Cancer Genomics Portal [34]. In the serous and endometrioid ECs within the TCGA cohort, copy number gains involving *TNK2* and *DDR1* were not focal but extended to flanking genes.

### A subset of *TNK2*and *DDR1*mutated tumors are *POLE*-mutant or microsatellite unstable

Somatic mutations in the exonuclease domain of *POLE* and/or microsatellite instability (MSI) occur in a subset of ECs and are associated with elevated mutation rates [21]. We therefore sought to determine whether any of the *TNK2-* or *DDR1*-mutated cases were coincident with *POLE* mutations or MSI-positivity. We sequenced exons 3–13 of *POLE*, which encode the exonuclease domain, from all 112 tumors in our study; the MSI status of tumors in this study has previously been reported [19]. Three tumors had somatically mutated *POLE* (T3 (c.C890T;p.S297F), T24 (c.1096delT; p.F367Lfs*15), and T97 (c.C857G; p.P286R), Additional file 9). Overall, somatic mutations within the exonuclease domain of *POLE* were detected in 2.2% (1 of 45) of serous, 4.8% (1 of 21) of clear cell, and 2.4% (1 of 41) of endometrioid tumors in our cohort. The frequency of *POLE* mutations in *TNK2*-*DDR1* mutated cases (1 of 7; 14%) compared with *TNK2*-*DDR1* non-mutated cases (2 of 105; 2%) was not statistically significantly different (*P* = 0.1775). Of the seven tumors with *TNK2* or *DDR1* mutations, one case (T3) had a somatic mutation within *POLE* (POLE^S297F^) and another three cases (T77, T88, and T117) were MSI-positive (Table 1). One of the two frameshift mutations in T117, an MSI-positive tumor, occurred at a polynucleotide (C_n_) tract (Additional file 6), suggesting that this mutation (TNK2^P761Rfs*72^) may have arisen as a consequence of defective mismatch repair.

## Discussion

Herein we report the occurrence of low-frequency somatic mutations in the *TNK2* and *DDR1* kinases among serous, clear cell, and endometrioid ECs. The TNK2 non-receptor tyrosine kinase is activated in response to a variety of stimuli including ligand-dependent stimulation of receptor tyrosine kinases [35], Cdc42 [36], and integrin-mediated cell adhesion [37]. TNK2 activation has been implicated in the regulation of cell growth, survival, and integrin-mediated cell adhesion and migration [37–41], and overexpression of TNK2 in cultured cells promotes a metastatic phenotype [37]. The DDR1 receptor tyrosine kinase is activated by triple-helical collagens [42] and has been implicated in the regulation of cell adhesion, survival, proliferation, differentiation, migration, invasion, morphogenesis and development [43–53].

Of the seven endometrial tumors that had somatic mutations in *TNK2* and/or *DDR1* in our study, one tumor (T3) was *POLE*-mutant and three tumors (T77, T88, and T117) were microsatellite-unstable, raising the possibility that the *TNK2* and *DDR1* mutations in these cases may have arisen as a consequence of replicative and mismatch repair defects respectively. A determination of whether the *TNK2* and *DDR1* mutations uncovered in this study are pathogenic driver mutations or incidental passenger mutations will ultimately rely on functional studies. In the interim, the potential effects of the *TNK2* and *DDR1* mutations on protein function can be postulated based on their positions relative to known functional domains of the encoded proteins and on *in silico* predictions. In this regard, the TNK2^R256H^ mutant occurs within the catalytic loop of TNK2, at a conserved residue that forms a hydrogen bond with an ATP analog [54], and is predicted, *in silico*, to impact protein function*.* The TNK2^D572N^ mutant occurs within a motif (LIDF) that is essential for binding to the clathrin heavy chain [55], and is predicted to be deleterious. Because a synthetic mutation (TNK2^D572A^) at this precise residue results in loss of clathrin binding [55], we speculate that the somatic TNK2^D572N^ mutant might likewise alter the TNK2-clathrin interaction. The TNK2^P761Rfs*72^ mutant was recurrent in our study occurring in two endometrioid ECs one of which was MSI-positive. The TNK2^P633Afs*3^ frameshift mutation may also be recurrent: we observed TNK2^P633Afs*3^ (chr3:195,595,228-195,595,229 insC; Hg19) in a *POLE*-mutant serous EC and this variant has been catalogued by others in cancer cell lines and tumors although in those instances it has not been subjected to technical validation (URL: http://www.cbioportal.org/public-portal/). Both TNK2^P633Afs*3^ and TNK2^P761Rfs*72^ are predicted to encode truncated forms of TNK2 that lack the UBA (ubiquitin associated) domain, which has been implicated in ligand-dependent proteasomal degradation of TNK2 [38, 56]. An earlier observation that deletion of the UBA domain of TNK2 results in elevated protein levels [56], together with a report that synthetic C-terminal deletion mutants of TNK2 retain catalytic activity [57, 58], raises the possibility that the naturally occurring TNK2^P633Afs*3^ and TNK2^P761Rfs*72^ mutants found in this study might encode elevated levels of truncated but catalytically active proteins.

The three *DDR1* mutations we identified in EC consisted of two missense mutations (DDR1^R570Q^ and DDR1^R574S^), and a frameshift mutation (DDR1^N740Ifs*10^) that occurred in an MSI-positive tumor. The DDR1^R570Q^ missense mutation, which we identified in a case of serous EC that was microsatellite-stable and *POLE*-wildtype, has been identified by others in an endometrioid EC [21], and in a case of metastatic melanoma [59]. Thus, the recurrent nature of the DDR1^R570Q^ mutation across studies suggests it may be a pathogenic event that provides a selective advantage in tumorigenesis, including endometrial tumorigenesis.

In the recent catalogue of genomic alterations reported by TCGA for endometrioid and serous ECs, somatic mutations of *TNK2* were documented in 2% of serous ECs and in 1% of endometrioid ECs, and somatic mutations of *DDR1* were noted in 4% of serous ECs and 2% of endometrioid ECs [21, 34, 60]. The eight mutations we uncovered in *TNK2* are different to the three *TNK2* mutations previously described in EC by TCGA. Similarly, two of the three mutations we describe in *DDR1* are unique to this study whereas, as discussed earlier, the third mutation (DDR1^R570Q^, CCDS4690; alternatively annotated as DDR1^R607Q^, CCDS34385) was present in a case of serous EC in this study and in a case of endometrioid EC by TCGA. Therefore, our observations not only validate the recent findings of low frequency somatic mutations in *TNK2* and *DDR1* in serous and endometrioid ECs by TCGA [21], but extend upon those findings by refining knowledge of the mutation spectrum of *TNK2* and *DDR1* in EC. Moreover, to our knowledge this is the first systematic search for somatic mutations in the tyrosine kinome of clear cell ECs.

In addition to somatic mutations, we also uncovered copy number gains involving *TNK2* at an appreciable frequency in serous and endometrioid ECs (8.9% and 2.4% respectively), and copy number gains involving *DDR1* at low frequency (2.2%) in serous ECs in our study. However, from an analysis of the TCGA endometrial cancer data, increased *TNK2* and *DDR1* copy number appears to reflect regional gains rather than focal amplification, thus making their potential biological relevance in endometrial cancer difficult to predict.

It is worth noting that our study has several limitations. First, our mutation discovery screen was restricted to the exons encoding the catalytic domains of tyrosine kinases and would not have detected mutations present in other exons. Second, our discovery screen did not have high statistical power to detect moderately to infrequently mutated genes (Additional file 5). Third, the use of Sanger sequencing for mutational analysis, in both the discovery screen and subsequent secondary screens of *TNK2* and *DDR1*, may have precluded the identification of sub-clonal variants that are below the sensitivity of detection by this methodology.

## Conclusions

In conclusion, we have identified rare somatic mutations and copy number alterations involving the *TNK2* and *DDR1* kinases amongst serous, clear cell, and endometrioid ECs. Our findings validate and extend the observation of *TNK2* and *DDR1* mutations in serous and endometrioid ECs catalogued by TCGA. To our knowledge, this is the first systematic search for somatic mutations in the tyrosine kinome of clear cell ECs. The recurrent nature of the TNK2^P761Rfs*72^ and DDR1^R570Q^ mutants raises the possibility that these may be pathogenic events that bestow a selective advantage in endometrial tumorigenesis. Future mechanistic studies of the somatic mutations reported herein are warranted.

### Availability of supporting data

All data supporting the somatic mutations reported in the manuscript are provided in Additional files 6, 7 and 9. Sanger sequencing files for the entire study will be made available through dbGAP with controlled access.

## Electronic supplementary material

Additional file 1:**Clinicopathological information for the mixed histology and endometrioid ECs in the study cohort.**(XLSX 47 KB)

Additional file 2:**PCR primers used in the discovery screen.**(XLSX 61 KB)

Additional file 3:**Tyrosine kinase genes analyzed in the mutation discovery screen.**(XLSX 14 KB)

Additional file 4:**PCR primers used in the secondary screens of** ***TNK2*** **and** ***DDR1.***(XLSX 14 KB)

Additional file 5:**Estimated statistical power to detect mutations in the discovery screen.**(XLSX 11 KB)

Additional file 6:**Sequence traces showing somatic mutations identified in** ***TNK2.*** Traces encompassing the mutated nucleotide (arrow) in tumor (T) DNA, and corresponding traces from matched normal (N) DNA are displayed. (ZIP 728 KB)

Additional file 7:**Sequence traces showing somatic mutations identified in** ***DDR1.*** Traces encompassing the mutated nucleotide (arrow) in tumor (T) DNA, and corresponding traces from matched normal (N) DNA are displayed. (ZIP 349 KB)

Additional file 8:**Oncoprints showing the distribution of somatic mutations and copy number alterations of** ***TNK2*** **and** ***DDR1*** **among 112 primary endometrial carcinomas in the discovery screen.** Individual tumors are displayed as gray bars; somatic mutations are indicated by dark blue bars; somatic copy number gains are indicated by green bars. The overall frequency (%) of somatic alterations for each histological subtype of endometrial cancer is shown on the right. (ZIP 517 KB)

Additional file 9:**Sequence traces showing somatic mutations identified in exons 3–13 of** ***POLE*** **, which encode the exonuclease domain of POLE.** Traces encompassing the mutated nucleotide (arrow) in tumor (T) DNA, and corresponding traces from matched normal (N) DNA are displayed. (ZIP 354 KB)

## Abbreviations

B2M: Beta-2-microglobulin
DDR1: Discoidin domain receptor tyrosine kinase 1
EC: Endometrial carcinoma
EECs: Endometrioid endometrial carcinomas
H&E: Hematoxylin and eosin
MSI: Microsatellite instability
POLE: Polymerase (DNA directed) epsilon catalytic subunit
TCGA: The cancer genome atlas
TNK2: Tyrosine kinase non-receptor 2
UBA: Ubiquitin associated.

## References

[1] Dedes KJ, Wetterskog D, Ashworth A, Kaye SB, Reis-Filho JS (2011). Emerging therapeutic targets in endometrial cancer. Nat Rev Clin Oncol.

[2] Mahboubi E, Eyler N, Wynder EL (1982). Epidemiology of cancer of the endometrium. Clin Obstet Gynecol.

[3] Dinkelspiel HE, Wright JD, Lewin SN, Herzog TJ (2013). Contemporary clinical management of endometrial cancer. Obstet Gynecol Int.

[4] Zighelboim I, Powell MA (2011). Adjuvant treatment for early-stage endometrial cancer. Clin Obstet Gynecol.

[5] Trope C, Kristensen GB, Abeler VM (2001). Clear-cell and papillary serous cancer: treatment options. Best Pract Res Clin Obstet Gynaecol.

[6] Hamilton CA, Cheung MK, Osann K, Chen L, Teng NN, Longacre TA, Powell MA, Hendrickson MR, Kapp DS, Chan JK (2006). Uterine papillary serous and clear cell carcinomas predict for poorer survival compared to grade 3 endometrioid corpus cancers. Brit J Cancer.

[7] Sherman ME (2000). Theories of endometrial carcinogenesis: a multidisciplinary approach. Mod Pathol.

[8] Webb GA, Lagios MD (1987). Clear cell carcinoma of the endometrium. Am J Obstet Gynecol.

[9] Setiawan VW, Yang HP, Pike MC, McCann SE, Yu H, Xiang YB, Wolk A, Wentzensen N, Weiss NS, Webb PM, van den Brandt PA, van de Vijver K, Thompson PJ, Strom BL, Spurdle AB, Soslow RA, Shu XO, Schairer C, Sacerdote C, Rohan TE, Robien K, Risch HA, Ricceri F, Rebbeck TR, Rastogi R, Prescott J, Polidoro S, Park Y, Olson SH, Moysich KB (2013). Type I and II endometrial cancers: have they different risk factors?. J Clin Oncol.

[10] del Carmen MG, Birrer M, Schorge JO (2012). Uterine papillary serous cancer: a review of the literature. Gynecol Oncol.

[11] Gadducci A, Cosio S, Spirito N, Cionini L (2010). Clear cell carcinoma of the endometrium: a biological and clinical enigma. Anticancer Res.

[12] Lynch TJ, Bell DW, Sordella R, Gurubhagavatula S, Okimoto RA, Brannigan BW, Harris PL, Haserlat SM, Supko JG, Haluska FG, Louis DN, Christiani DC, Settleman J, Haber DA (2004). Activating mutations in the epidermal growth factor receptor underlying responsiveness of non-small-cell lung cancer to gefitinib. N Engl J Med.

[13] Druker BJ, Sawyers CL, Kantarjian H, Resta DJ, Reese SF, Ford JM, Capdeville R, Talpaz M (2001). Activity of a specific inhibitor of the BCR-ABL tyrosine kinase in the blast crisis of chronic myeloid leukemia and acute lymphoblastic leukemia with the Philadelphia chromosome. N Engl J Med.

[14] Druker BJ, Talpaz M, Resta DJ, Peng B, Buchdunger E, Ford JM, Lydon NB, Kantarjian H, Capdeville R, Ohno-Jones S, Sawyers CL (2001). Efficacy and safety of a specific inhibitor of the BCR-ABL tyrosine kinase in chronic myeloid leukemia. N Engl J Med.

[15] Paez JG, Janne PA, Lee JC, Tracy S, Greulich H, Gabriel S, Herman P, Kaye FJ, Lindeman N, Boggon TJ, Naoki K, Sasaki H, Fujii Y, Eck MJ, Sellers WR, Johnson BE, Meyerson M (2004). EGFR mutations in lung cancer: correlation with clinical response to gefitinib therapy. Science.

[16] Kwak EL, Bang YJ, Camidge DR, Shaw AT, Solomon B, Maki RG, Ou SH, Dezube BJ, Janne PA, Costa DB, Varella-Garcia M, Kim WH, Lynch TJ, Fidias P, Stubbs H, Engelman JA, Sequist LV, Tan W, Gandhi L, Mino-Kenudson M, Wei GC, Shreeve SM, Ratain MJ, Settleman J, Christensen JG, Haber DA, Wilner K, Salgia R, Shapiro GI, Clark JW (2010). Anaplastic lymphoma kinase inhibition in non-small-cell lung cancer. N Engl J Med.

[17] Weinstein IB (2002). Cancer. Addiction to oncogenes–the Achilles heal of cancer. Science.

[18] Dutt A, Salvesen HB, Chen TH, Ramos AH, Onofrio RC, Hatton C, Nicoletti R, Winckler W, Grewal R, Hanna M, Wyhs N, Ziaugra L, Richter DJ, Trovik J, Engelsen IB, Stefansson IM, Fennell T, Cibulskis K, Zody MC, Akslen LA, Gabriel S, Wong KK, Sellers WR, Meyerson M, Greulich H (2008). Drug-sensitive FGFR2 mutations in endometrial carcinoma. Proc Natl Acad Sci U S A.

[19] Le Gallo M, O'Hara AJ, Rudd ML, Urick ME, Hansen NF, O'Neil NJ, Price JC, Zhang S, England BM, Godwin AK, Sgroi DC, Hieter P, Mullikin JC, Merin MJ, Bell DW (2012). Exome sequencing of serous endometrial tumors identifies recurrent somatic mutations in chromatin-remodeling and ubiquitin ligase complex genes. Nat Genet.

[20] Liang H, Cheung LW, Li J, Ju Z, Yu S, Stemke-Hale K, Dogruluk T, Lu Y, Liu X, Gu C, Guo W, Scherer SE, Carter H, Westin SN, Dyer MD, Verhaak RG, Zhang F, Karchin R, Liu CG, Lu KH, Broaddus RR, Scott KL, Hennessy BT, Mills GB (2012). Whole-exome sequencing combined with functional genomics reveals novel candidate driver cancer genes in endometrial cancer. Genome Res.

[21] Kandoth C, Schultz N, Cherniack AD, Akbani R, Liu Y, Shen H, Robertson AG, Pashtan I, Shen R, Benz CC, Yau C, Laird PW, Ding L, Zhang W, Mills GB, Kucherlapati R, Mardis ER, Levine DA, The Cancer Genome Atlas Research Network (2013). Integrated genomic characterization of endometrial carcinoma. Nature.

[22] Kinde I, Bettegowda C, Wang Y, Wu J, Agrawal N, Shih Ie M, Kurman R, Dao F, Levine DA, Giuntoli R, Roden R, Eshleman JR, Carvalho JP, Marie SK, Papadopoulos N, Kinzler KW, Vogelstein B, Diaz LA (2013). Evaluation of DNA from the Papanicolaou test to detect ovarian and endometrial cancers. Sci Transl Med.

[23] Kuhn E, Wu RC, Guan B, Wu G, Zhang J, Wang Y, Song L, Yuan X, Wei L, Roden RB, Kuo KT, Nakayama K, Clarke B, Shaw P, Olvera N, Kurman RJ, Levine DA, Wang TL, Shih Ie M (2012). Identification of molecular pathway aberrations in uterine serous carcinoma by genome-wide analyses. J Natl Cancer Inst.

[24] Zhao S, Choi M, Overton JD, Bellone S, Roque DM, Cocco E, Guzzo F, English DP, Varughese J, Gasparrini S, Bortolomai I, Buza N, Hui P, Abu-Khalaf M, Ravaggi A, Bignotti E, Bandiera E, Romani C, Todeschini P, Tassi R, Zanotti L, Carrara L, Pecorelli S, Silasi DA, Ratner E, Azodi M, Schwartz PE, Rutherford TJ, Stiegler AL, Mane S (2013). Landscape of somatic single-nucleotide and copy-number mutations in uterine serous carcinoma. Proc Natl Acad Sci U S A.

[25] Hudson TJ, Anderson W, Artez A, Barker AD, Bell C, Bernabe RR, Bhan MK, Calvo F, Eerola I, Gerhard DS, Guttmacher A, Guyer M, Hemsley FM, Jennings JL, Kerr D, Klatt P, Kolar P, Kusada J, Lane DP, Laplace F, Youyong L, Nettekoven G, Ozenberger B, Peterson J, Rao TS, Remacle J, Schafer AJ, Shibata T, Stratton MR, Vockley JG (2010). International network of cancer genome projects. Nature.

[26] Chines P, Swift A, Bonnycastle LL, Erdos MR, Mullikin J, Collins FS, NISC (2005). PrimerTile: designing overlapping PCR primers for resequencing. Am J Hum Genet.

[27] Rice JC, Pollock LM, Rudd ML, Fogoros SK, Mohamed H, Hanigan CL, Le Gallo M, Program NI, Zhang S, Cruz P, Cherukuri PF, Hansen NF, McManus KJ, Godwin AK, Sgroi DC, Mullikin JC, Merino MJ, Hieter P, Bell DW (2013). Sequencing of candidate chromosome instability genes in endometrial cancers reveals somatic mutations in ESCO1, CHTF18, and MRE11A. PLoS One.

[28] Way DL, Grosso DS, Davis JR, Surwit EA, Christian CD (1983). Characterization of a new human endometrial carcinoma (RL95-2) established in tissue culture. In vitro.

[29] Richardson GS, Dickersin GR, Atkins L, MacLaughlin DT, Raam S, Merk LP, Bradley FM (1984). KLE: a cell line with defective estrogen receptor derived from undifferentiated endometrial cancer. Gynecol Oncol.

[30] Kurarmoto H, Hamano M, Imai M (2002). HEC-1 cells. Hum Cell.

[31] Satyaswaroop PG, Fleming H, Bressler RS, Gurpide E (1978). Human endometrial cancer cell cultures for hormonal studies. Cancer Res.

[32] Kuramoto H (1972). Studies of the growth and cytogenetic properties of human endometrial adenocarcinoma in culture and its development into an established line. Acta Obstet Gynaecol Jpn.

[33] Davies H, Bignell GR, Cox C, Stephens P, Edkins S, Clegg S, Teague J, Woffendin H, Garnett MJ, Bottomley W, Davis N, Dicks E, Ewing R, Floyd Y, Gray K, Hall S, Hawes R, Hughes J, Kosmidou V, Menzies A, Mould C, Parker A, Stevens C, Watt S, Hooper S, Wilson R, Jayatilake H, Gusterson BA, Cooper C, Shipley J (2002). Mutations of the BRAF gene in human cancer. Nature.

[34] Cerami E, Gao J, Dogrusoz U, Gross BE, Sumer SO, Aksoy BA, Jacobsen A, Byrne CJ, Heuer ML, Larsson E, Antipin Y, Reva B, Goldberg AP, Sander C, Schultz N (2012). The cBio cancer genomics portal: an open platform for exploring multidimensional cancer genomics data. Cancer Discov.

[35] Galisteo ML, Yang Y, Urena J, Schlessinger J (2006). Activation of the nonreceptor protein tyrosine kinase Ack by multiple extracellular stimuli. Proc Natl Acad Sci U S A.

[36] Manser E, Leung T, Salihuddin H, Tan L, Lim L (1993). A non-receptor tyrosine kinase that inhibits the GTPase activity of p21cdc42. Nature.

[37] van der Horst EH, Degenhardt YY, Strelow A, Slavin A, Chinn L, Orf J, Rong M, Li S, See LH, Nguyen KQ, Hoey T, Wesche H, Powers S (2005). Metastatic properties and genomic amplification of the tyrosine kinase gene ACK1. Proc Natl Acad Sci U S A.

[38] Shen F, Lin Q, Gu Y, Childress C, Yang W (2007). Activated Cdc42-associated kinase 1 is a component of EGF receptor signaling complex and regulates EGF receptor degradation. Mol Biol Cell.

[39] Lin Q, Wang J, Childress C, Sudol M, Carey DJ, Yang W (2010). HECT E3 ubiquitin ligase Nedd4-1 ubiquitinates ACK and regulates epidermal growth factor (EGF)-induced degradation of EGF receptor and ACK. Mol Cell Biol.

[40] Modzelewska K, Newman LP, Desai R, Keely PJ (2006). Ack1 mediates Cdc42-dependent cell migration and signaling to p130Cas. J Biol Chem.

[41] Mahajan K, Mahajan NP (2010). Shepherding AKT and androgen receptor by Ack1 tyrosine kinase. J Cell Physiol.

[42] Vogel W (1999). Discoidin domain receptors: structural relations and functional implications. FASEB J.

[43] Curat CA, Vogel WF (2002). Discoidin domain receptor 1 controls growth and adhesion of mesangial cells. J Am Soc Nephrol.

[44] Yeh YC, Wu CC, Wang YK, Tang MJ (2011). DDR1 triggers epithelial cell differentiation by promoting cell adhesion through stabilization of E-cadherin. Mol Biol Cell.

[45] Valencia K, Ormazabal C, Zandueta C, Luis-Ravelo D, Anton I, Pajares MJ, Agorreta J, Montuenga LM, Martinez-Canarias S, Leitinger B, Lecanda F (2012). Inhibition of collagen receptor discoidin domain receptor-1 (DDR1) reduces cell survival, homing, and colonization in lung cancer bone metastasis. Clin Cancer Res.

[46] Wang CZ, Hsu YM, Tang MJ (2005). Function of discoidin domain receptor I in HGF-induced branching tubulogenesis of MDCK cells in collagen gel. J Cell Physiol.

[47] Kim HG, Hwang SY, Aaronson SA, Mandinova A, Lee SW (2011). DDR1 receptor tyrosine kinase promotes prosurvival pathway through Notch1 activation. J Biol Chem.

[48] Hou G, Vogel W, Bendeck MP (2001). The discoidin domain receptor tyrosine kinase DDR1 in arterial wound repair. J Clin Invest.

[49] Hidalgo-Carcedo C, Hooper S, Chaudhry SI, Williamson P, Harrington K, Leitinger B, Sahai E (2011). Collective cell migration requires suppression of actomyosin at cell-cell contacts mediated by DDR1 and the cell polarity regulators Par3 and Par6. Nat Cell Biol.

[50] Hou G, Vogel WF, Bendeck MP (2002). Tyrosine kinase activity of discoidin domain receptor 1 is necessary for smooth muscle cell migration and matrix metalloproteinase expression. Circ Res.

[51] Kamohara H, Yamashiro S, Galligan C, Yoshimura T (2001). Discoidin domain receptor 1 isoform-a (DDR1alpha) promotes migration of leukocytes in three-dimensional collagen lattices. FASEB J.

[52] Bhatt RS, Tomoda T, Fang Y, Hatten ME (2000). Discoidin domain receptor 1 functions in axon extension of cerebellar granule neurons. Gene Dev.

[53] Vogel WF, Aszodi A, Alves F, Pawson T (2001). Discoidin domain receptor 1 tyrosine kinase has an essential role in mammary gland development. Mol Cell Biol.

[54] Lougheed JC, Chen RH, Mak P, Stout TJ (2004). Crystal structures of the phosphorylated and unphosphorylated kinase domains of the Cdc42-associated tyrosine kinase ACK1. J Biol Chem.

[55] Teo M, Tan L, Lim L, Manser E (2001). The tyrosine kinase ACK1 associates with clathrin-coated vesicles through a binding motif shared by arrestin and other adaptors. J Biol Chem.

[56] Chan W, Tian R, Lee YF, Sit ST, Lim L, Manser E (2009). Down-regulation of active ACK1 is mediated by association with the E3 ubiquitin ligase Nedd4-2. J Biol Chem.

[57] Prieto-Echague V, Gucwa A, Brown DA, Miller WT (2010). Regulation of Ack1 localization and activity by the amino-terminal SAM domain. BMC Biochem.

[58] Chan W, Sit ST, Manser E (2011). The Cdc42-associated kinase ACK1 is not autoinhibited but requires Src for activation. Biochem J.

[59] Wei X, Walia V, Lin JC, Teer JK, Prickett TD, Gartner J, Davis S, Stemke-Hale K, Davies MA, Gershenwald JE, Robinson W, Robinson S, Rosenberg SA, Samuels Y (2011). Exome sequencing identifies GRIN2A as frequently mutated in melanoma. Nat Genet.

[60] Gao J, Aksoy BA, Dogrusoz U, Dresdner G, Gross B, Sumer SO, Sun Y, Jacobsen A, Sinha R, Larsson E, Cerami E, Sander C, Schultz N (2013). Integrative analysis of complex cancer genomics and clinical profiles using the cBioPortal. Sci Signal.

[CR61] The pre-publication history for this paper can be accessed here:http://www.biomedcentral.com/1471-2407/14/884/prepub

